# Basal Forebrain Atrophy Contributes to Allocentric Navigation Impairment in Alzheimer’s Disease Patients

**DOI:** 10.3389/fnagi.2015.00185

**Published:** 2015-09-28

**Authors:** Georg M. Kerbler, Zuzana Nedelska, Jurgen Fripp, Jan Laczó, Martin Vyhnalek, Jiří Lisý, Adam S. Hamlin, Stephen Rose, Jakub Hort, Elizabeth J. Coulson

**Affiliations:** ^1^Clem Jones Centre for Ageing Dementia Research, Queensland Brain Institute, The University of Queensland, Brisbane, QLD, Australia; ^2^Memory Clinic, Department of Neurology, 2nd Faculty of Medicine, Charles University in Prague and Motol University Hospital, Prague, Czech Republic; ^3^International Clinical Research Center, St. Anne’s University Hospital Brno, Brno, Czech Republic; ^4^Computational Informatics, Commonwealth Scientific and Industrial Research Organisation, Brisbane, QLD, Australia; ^5^Department of Radiology, 2nd Faculty of Medicine, Charles University in Prague and Motol University Hospital, Prague, Czech Republic

**Keywords:** basal forebrain, navigation, Alzheimer’s disease, MRI, cognitive impairment

## Abstract

The basal forebrain degenerates in Alzheimer’s disease (AD) and this process is believed to contribute to the cognitive decline observed in AD patients. Impairment in spatial navigation is an early feature of the disease but whether basal forebrain dysfunction in AD is responsible for the impaired navigation skills of AD patients is not known. Our objective was to investigate the relationship between basal forebrain volume and performance in real space as well as computer-based navigation paradigms in an elderly cohort comprising cognitively normal controls, subjects with amnestic mild cognitive impairment and those with AD. We also tested whether basal forebrain volume could predict the participants’ ability to perform allocentric- vs. egocentric-based navigation tasks. The basal forebrain volume was calculated from 1.5 T magnetic resonance imaging (MRI) scans, and navigation skills were assessed using the human analog of the Morris water maze employing allocentric, egocentric, and mixed allo/egocentric real space as well as computerized tests. When considering the entire sample, we found that basal forebrain volume correlated with spatial accuracy in allocentric (cued) and mixed allo/egocentric navigation tasks but not the egocentric (uncued) task, demonstrating an important role of the basal forebrain in mediating cue-based spatial navigation capacity. Regression analysis revealed that, although hippocampal volume reflected navigation performance across the entire sample, basal forebrain volume contributed to mixed allo/egocentric navigation performance in the AD group, whereas hippocampal volume did not. This suggests that atrophy of the basal forebrain contributes to aspects of navigation impairment in AD that are independent of hippocampal atrophy.

## Introduction

The basal forebrain, encompassing the septum, vertical and horizontal diagonal bands of Broca, and the nucleus basalis of Meynert, degenerates in Alzheimer’s disease (AD), and this process plays a role in the general cognitive decline observed in AD patients (Mufson, 2003; Schliebs and Arendt, 2006). Using magnetic resonance imaging (MRI), smaller basal forebrain volumes have been shown to be associated with poorer performances in a multitude of cognitive tests used to measure memory dysfunction in AD, including the clinical dementia rating (George et al., 2011; Grothe et al., 2012) and the California verbal learning test (Butler et al., 2012), as well as the mini-mental state examination (MMSE; Grothe et al., 2011).

Basal forebrain neurons (e.g., cholinergic neurons), which project to the hippocampus, amygdala, and almost the entire neocortex, have also been reported to play a role in attention in humans (Hasselmo and McGaughy, 2004; Sarter et al., 2006), and lesion studies in rodents have demonstrated that selective loss of basal forebrain cholinergic neurons causes impairment in both egocentric (uncued) and allocentric (cued) spatial navigation (Berger-Sweeney et al., 2001; Hamlin et al., 2013). The basal forebrain cholinergic system has also been shown to be involved in feature binding – the ability to process different features of objects and combine these into a complete picture (Botly and De Rosa, 2009) – and visuospatial attention (Botly and De Rosa, 2012), which together are crucial for correct allocentric navigation.

It is known that acetylcholine is released from basal forebrain neurons when spatial cues are detected, but not when cues are missed (Parikh et al., 2007). Likewise, the administration of donepezil, an acetylcholine esterase inhibitor that is used to treat cognitive dysfunction in patients diagnosed with mild AD by prolonging synaptic acetylcholine availability, has been shown to improve the allocentric navigation performance of patients in a human analog of the Morris water maze (hMWM; Hort et al., 2014), a commonly used spatial working memory task. Taken together, these studies suggest a role for basal forebrain function in allocentric navigation; however, the role of the basal forebrain in allocentric vs. egocentric spatial navigation has never been studied *in vivo* in humans.

Here, we investigated the relationship between basal forebrain volume (measured by MRI) and performance in cued (allocentric), uncued (egocentric), and mixed allo/egocentric spatial navigation in the real space and computerized versions of the hMWM task. We also assessed differences in basal forebrain volume between groups and determined whether impaired performance in hMWM tasks in patients with amnestic mild cognitive impairment (aMCI) and AD was associated with basal forebrain volume.

## Materials and Methods

### Subjects and eligibility

Between October 2010 and October 2012, patients (Table 1) with probable AD (*n* = 22) or aMCI (*n* = 27) were consecutively recruited and followed up at the Memory Disorders Clinic, Department of Neurology, 2nd Medical Faculty of Charles University and Motol University Hospital in Prague, the Czech Republic (a longitudinal memory clinic-based cohort). Patients were originally referred to the memory clinic by general practitioners, local neurologists, psychiatrists, and geriatricians based on memory complaint from the patient or the proxy. For comparison, cognitively normal elderly who we refer to as normal controls (NC; *n* = 17) were recruited from the University of the Third Age, 2nd Medical Faculty of Charles University in Prague, or patients’ family members. All aforementioned subjects met the eligibility criteria defined as the absence of primary neurological or psychiatric disease other than aMCI and AD that could interfere with cognitive functioning and sufficient brain scan quality free of structural abnormalities such as tumors, cortical infarcts, subdural hematomas, or hydrocephalus, whereas lacunar infarcts and white matter (WM) hyperintensities were not excluded. The subjects or their proxies signed an informed consent, and the study was approved by the joint Institutional Review Board of the 2nd Medical Faculty, Charles University in Prague and Motol University Hospital.

**Table 1:** **Demographics of NC, aMCI, and AD groups**.

	NC	aMCI	AD
	(*n* = 17)	(*n* = 27)	(*n* = 22)
Age	70.1 ± 4.8	71.4 ± 8.1	74.7 ± 8.1
Gender M/F	7/10	17/10	10/12
Years of education	15.6 ± 2.3	15.1 ± 3.2	12.1 ± 2.3
MMSE	28.5 ± 1.7	25.3 ± 3.8	19.5 ± 2.9

*Age and years of education are expressed as mean ± SD*.

### Clinical evaluations and diagnosis

Subjects received clinical, neurological, internal and laboratory evaluations, neuropsychological examination, brain MRI scans, and spatial navigation tests using the hMWM. Diagnosis was assigned to the subjects during a panel consensus conference of three neurologists working in the field of neurodegerative diseases and dementia each having 10 or more years of clinical experience using patients’ clinical status, neuropsychology scores, brain scans, and accepted diagnostic criteria.

Subjects with AD met the DSM-IV-TR criteria for dementia and the National Institute of Neurological and Communicative Diseases and Stroke/Alzheimer’s Disease and Related Disorders Association criteria for probable AD (McKhann et al., 1984).

Subjects with aMCI met criteria for aMCI (Petersen, 2004), including memory complaints reported by a patient or a proxy, evidence of memory dysfunction on neuropsychological examination, intact activities of daily living and absence of dementia. aMCI subjects scored no greater than 0.5 on Clinical Dementia Rating scale (global score), which commonly designates MCI (Morris, 1993). Memory impairment was established when the subject scored more than 1.5 SDs below the mean of age- and education-adjusted norms on any memory test (Laczo et al., 2011). The aMCI group included both those with isolated memory impairment (single-domain aMCI) and those with memory and an additional impairment in any other non-memory domain (multiple-domain aMCI). Subjects were diagnosed as cognitively normal when they reported no subjective cognitive complaints and scored within 1.5 SDs from the mean of age- and education-adjusted normative on any cognitive test.

### Neuropsychological examination

The neuropsychological battery comprised the following tests: 1. Verbal memory measured using the Rey Auditory Verbal Learning Test trials 1–5, the Rey Auditory Verbal Learning Test 30-min Delayed Recall trial, and a 16-item picture version of the Enhanced Cued Recall Test (free and total recall scores); 2. Non-verbal memory measured using the Rey–Osterrieth Complex Figure Test (the Immediate Recall condition); 3. Visuospatial function measured using the Rey-Osterrieth Complex Figure Test (the Copy condition); 4. Executive function measured using the Trail Making Test B and Controlled Oral Word Association Test; 5. Attention and working memory measured using the Forward and Backward Digit Span and Trail Making Test A; and 6. Language measured using the Boston Naming Test (30-item version). The MMSE measured global cognitive function.

### Spatial navigation testing using the human analog of the morris water maze

**Figure 1:**
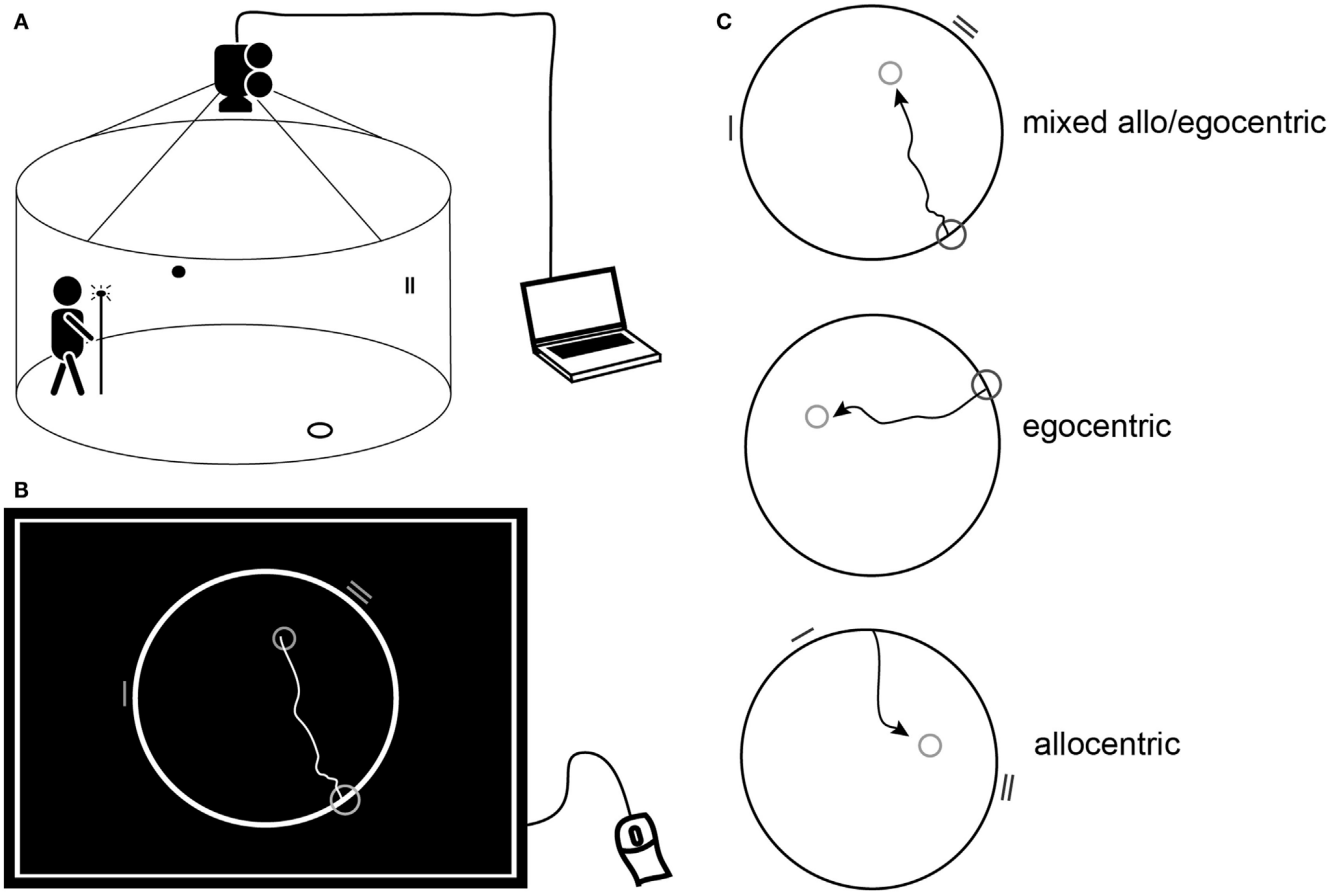
**Human analog of the MWM**. **(A)** Schematic diagram of the real space version of the human MWM indicating the circular target zone on the floor, wall cues within the arena, and the lit pole used to indicate the position of the target. **(B)** Schematic of a computer screen view displaying the computerized task. The largest circle represents the arena, the smallest circle in the arena representing the goal position, the mid-sized circle on the edge of the arena representing the start position, and the lines on the edge of the arena representing the visual cues. The trajectory shows an individual subject’s hypothetical solution of getting from the start to the goal. **(B,C)** Schematic of the three subtasks: mixed allo/egocentric, egocentric (uncued), and allocentric (cued) displayed as for the computerized task or as an above view of the real space arena.

Spatial navigation was tested using in-house developed real space and computerized versions of the hMWM (Figure 1) based in the Laboratory of Spatial Cognition, a joint workplace of the Department of Neurology, 2nd Faculty of Medicine, Charles University and the Institute of Physiology, Academy of Sciences of the Czech Republic. The hMWM was designed to examine the two basic types of navigation: (1) allocentric (cued) navigation, which is hippocampus-driven, self-position-independent, and uses salient distal cues to find the goal (Astur et al., 2002) and (2) egocentric (uncued) navigation, which is predominantly parietal cortex- and caudate nucleus-driven, and is self- and start-position-dependent (Weniger et al., 2009). The real space and computer-based versions of the task are described in detail elsewhere (Hort et al., 2007; Laczo et al., 2011).

Briefly, the real space task was conducted within a fully enclosed arena surrounded by a dark blue velvet curtain and operated by a computer program. The computer-based 2D task was conducted using the map-view of the velvet arena represented by a circle on a computer touch screen. The purpose of the task, regardless of the version used, was to locate the invisible goal on the arena floor or within the circle on the computer screen using either the start position (uncued, egocentric), two distal orientation cues (cued, allocentric), or both the start position and orientation cues (mixed allo/egocentric). The (invisible) goal was initially shown to subjects for approximately 10–15 s immediately prior to the first trial. In the computer-based version, subjects were asked to use a mouse and screen to draw the way from the start to the goal. In the real space version, they were asked to walk directly from the start, and to indicate exactly where they thought the goal was by pointing a long stick with a light-emitting diode to the floor once they walked into goal’s presumed position.

Navigation performance was recorded automatically based on the position of either the point drawn by the patient on the computer screen or the diode on the stick location. The navigation accuracy was measured as the distance error (in pixels in the computerized version and in centimeters in the real space version) between the goal position determined by the subject, and the correct goal position. Each of the allocentric, egocentric, and mixed tasks consisted of eight trials in both versions. Each computerized task preceded the respective real space task. The order of the tasks was as follows: (1) mixed allo/egocentric, (2) egocentric, and (3) allocentric. Trials had no time limit, mainly to reduce bias caused by differences in cognitive, sensory, and physical functioning. Examiners were blinded to the diagnosis and supervised the task without interference beyond standard instructions.

### MRI acquisition

Images were acquired at 1.5 T (Siemens AG, Erlangen, Germany) using T1-weighted 3-dimensional high-resolution magnetization-prepared rapid acquisition with gradient echo (MPRAGE) sequence with the following parameters: TR/TE/TI = 2000/3.08/1100 ms, flip angle 15°, 192 continuous partitions, slice thickness 1.0 mm, and in-plane resolution 1 mm. Scans were visually examined by a single neuroradiologist, blinded to the diagnosis, for sufficient technical quality and the absence of structural findings contradicting eligibility.

### MRI processing

The hippocampus, pons and gray matter (GM), WM, and cerebrospinal fluid (CSF) were segmented from the MPRAGE images using the method outlined in Bourgeat et al. (2010). A skull strip mask was generated from the GM, WM, and CSF segmentation. All volume calculations reported are normalized by intracranial volume.

The skull-stripped MPRAGE images of 80 healthy elderly subjects from the Australian Imaging Biomarkers and Lifestyle study (AIBL; http://www.aibl.csiro.au/) were normalized to create an average elderly template brain using the open-source deformable registration tool ANTS (Avants et al., 2010). The population-specific template was generated by iteratively registering images to the current template estimate. A new shape and intensity average were then computed from the results of the registrations and the current template estimate was set as the average. This template generation procedure was repeated iteratively (*I* = 5). Each registration in this procedure used the greedy symmetric normalization algorithm (SyN, parameters outlined below; Avants et al., 2008) and the cross-correlation after subtracting the local mean from the image match metric.

The registration between each subject’s MPRAGE image and the population-specific template was performed using the SyN algorithm (GradStep = 0.5, regularization sigma = 2.0). The image match metric was the cross-correlation between the images. Cross-correlation after subtracting the local mean from the image at each voxel was computed using a 5 × 5 × 5 voxel window. Registration was performed in a multi-resolution scheme, with a maximum of 30 iterations at 4× subsampling, 90 iterations at 2× subsampling, and 50 iterations at full resolution.

We have previously established a mask of the entire basal forebrain, encompassing only those areas that undergo atrophy in AD patients, by comparing control (*n* = 80) and AD (*n* = 38) subjects from the AIBL cohort (Kerbler et al., 2014) and overlaid the resultant *z*-score map (set to −0.5 and −1 SDs) on a standard Montreal Neurological Institute (MNI) brain. Using published probabilistic basal forebrain maps derived from histological data as a guide for the limits of the structure (Zaborszky et al., 2008), regions of atrophy within the standard space corresponding to the basal forebrain were then segmented.

We used maximum probability maps (MPMs; Zaborszky et al., 2008) as guidance, resulting in a mask covering voxels that were identified as lying in the basal forebrain in 10 post-mortem brains and a final delineated area of high anatomical specificity. To study anterior and posterior basal forebrain compartments separately, we further divided the basal forebrain map into an anterior volume (size: 595 voxels), covering the medial septum and diagonal bands of Broca (Ch1–3) as well as anterior regions of the nucleus basalis of Meynert (Ch4), and a posterior volume (size: 565 voxels), covering the intermediate and posterior nucleus basalis of Meynert (nomenclature according to Zaborszky et al., 2008). Note, that basal forebrain MRI maps used by us and other researchers are not specific for cholinergic cells of the basal forebrain, as basal forebrain nuclei also contain other neuronal subtypes (e.g., GABAergic projection neurons). The masks were propagated to the subjects’ space to extract the basal forebrain volume, and GM and WM masks were used to remove any falsely included CSF voxels.

### Statistics

Basal forebrain and hippocampal volumes as well as navigation scores were corrected for age, education, and gender. Analysis of variance (ANOVA) in conjunction with Bonferroni *post hoc* testing was used to calculate group differences. The *p*-values given are the corrected values. Two-tailed Pearson’s product moment correlation between age-, education-, and gender-corrected variables (volumes and navigation scores) was used. Multiple linear regressions were performed to assess the relationships between modalities in predicting distance error in navigation tests. The actual *p*-values are provided except where *p* was less than 0.001, which is written as *p* < 0.001. *p* < 0.05 was considered as significant.

## Results

### Basal forebrain volumes are significantly reduced in aMCI

**Figure 2:**
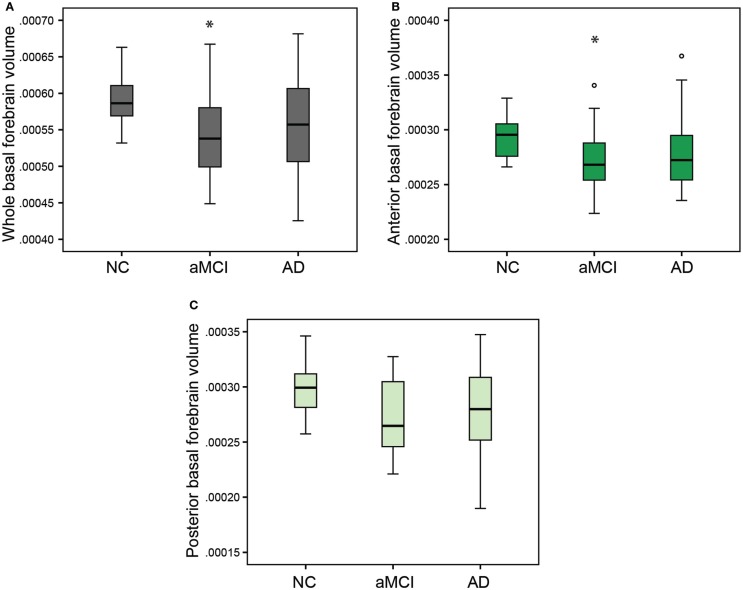
**Group comparisons of whole, anterior, and posterior basal forebrain volumes between the NC, aMCI, and AD groups**. There was a significant reduction in whole **(A)** and anterior **(B)** but not posterior **(C)** basal forebrain volume between the NC and aMCI groups. The AD group was not significantly different from either the NC or aMCI group for any of the basal forebrain volumes. Whiskers represent min/max values except for data points (circles) more than 1.5 interquartile ranges away from the 75th percentile. *p < 0.05

**Figure 3:**
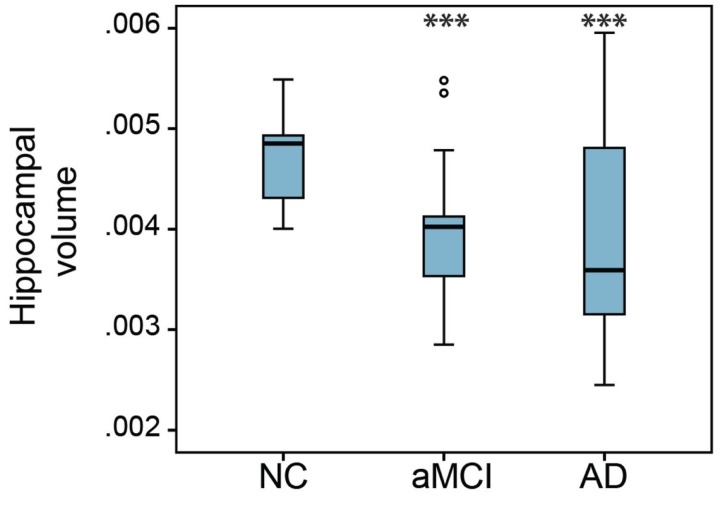
**Group comparison of hippocampal volumes between NC, aMCI, and AD groups**. There was a significant reduction of hippocampal volume in the aMCI and AD groups compared to the NC group. The hippocampal volume of the AD group was not significantly different from that of the aMCI group. Whiskers represent min/max values except for data points (circles) more than 1.5 interquartile ranges away from the 75th percentile. ***p < 0.001

In order to compare the basal forebrain integrity between clinical groups, we first calculated the basal forebrain volume of the whole structure, as well as the anterior and the posterior regions. These volumes were then corrected for age, education, and gender, and subsequently compared between the NC, aMCI, and AD groups, as well as being correlated to the hippocampal volume. Group comparisons revealed that the mean whole (Figure 2A; *p* = 0.032), as well as the anterior (Figure 2B; *p* = 0.044), but not the posterior (Figure 2C) basal forebrain volumes were significantly reduced in aMCI subjects compared to NC. Hippocampal volumes were significantly reduced in the aMCI and AD subject groups (Figure 3). Although we and other groups have reported significant reductions in BF volumes for AD groups previously (Grothe et al., 2011; Kerbler et al., 2014), this was not the case for the current cohort. No other significant differences in mean basal forebrain volume were found between diagnostic groups, perhaps due to the small group sizes (Table 2). As found previously (Kerbler et al., 2014), the whole (*p* < 0.001), anterior (*p* < 0.001), and posterior (*p* < 0.001) basal forebrain volumes were highly correlated to the hippocampal volume.

**Table 2:** **Mean volumes of basal forebrain (anterior, posterior, and whole) and hippocampus of NC, aMCI, and AD groups**.

	NC	aMCI	AD
BF anterior	29.39 ± 1.85	27.14 ± 2.92*	27.92 ± 3.43
BF posterior	29.75 ± 2.46	27.18 ± 3.55	27.78 ± 4.20
BF whole	59.14 ± 3.73	54.32 ± 6.09*	55.70 ± 6.95
Hippocampus	474.78 ± 65.00	391.56 ± 65.00**	387.25 ± 96.09**

*Significantly different from NC (at   0.05).

**Significantly different from NC (at   0.01).

*Volumes were corrected for age, gender, and years of education and are expressed as mean ± SD (in mm^3^ ×10^−5^)*.

### Basal forebrain volume correlates with navigation scores in the whole cohort

**Figure 4:**
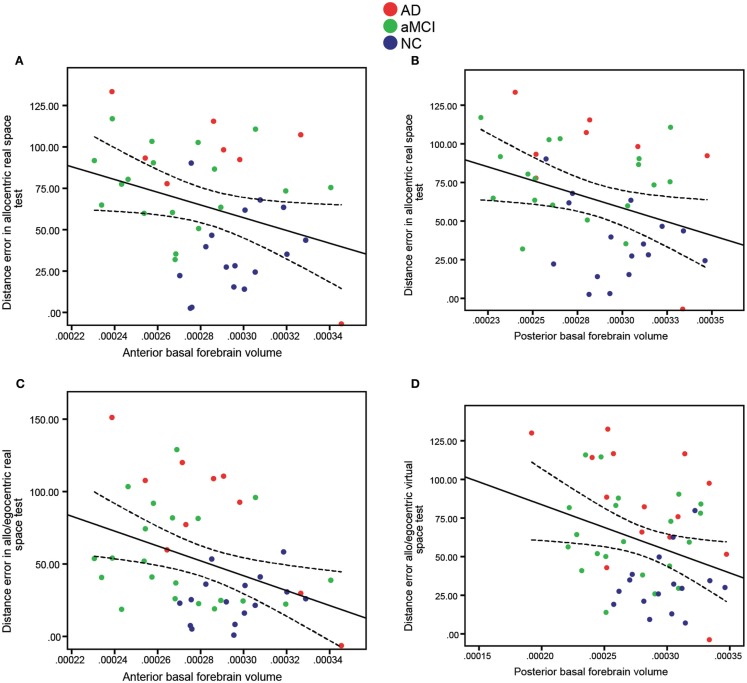
**Significant correlations of anterior and posterior basal forebrain volumes (*x*-axis) to distance error (*y*-axis) in allocentric (A,B) and mixed allo/egocentric (C,D) real space tests**. Both **(A)** Anterior (*R* = 0.32, *p* = 0.041) and **(B)** Posterior (*R* = 0.34, *p* = 0.028) basal forebrain volumes were significantly correlated to distance error in the allocentric real space test. Furthermore, distance error in the mixed allo/egocentric real space test was correlated to **(C)** Anterior basal forebrain volume (*R* = 0.38, *p* = 0.009), whereas distance error in the mixed allo/egocentric virtual space test was correlated to **(D)** Posterior basal forebrain volume (*R* = 0.31, *p* = 0.027). Data points are colored according to clinical diagnosis. A linear fit line with 95% confidence interval is shown.

To investigate the relationship between allocentric, egocentric, as well as mixed allo/egocentric spatial navigation performance and the basal forebrain, we correlated navigation scores of both real space and computer-based versions of the navigation tasks with basal forebrain volumes of the elderly and cognitively impaired cohorts. In the whole cohort (Figure 4; Table 3), the distance error in the allocentric real space task was correlated to the anterior (Figure 4A; *R* = 0.32, *p* = 0.041), posterior (Figure 4B; *R* = 0.34, *p* = 0.028), and whole (*R* = 0.35, *p* = 0.022) basal forebrain volume. Furthermore, distance error in the mixed allo/egocentric real space test was significantly associated with anterior (Figure 4C; *R* = 0.38, *p* = 0.009) and whole (*R* = 0.34, *p* = 0.019) but not posterior basal forebrain volume. Associations between computer-based navigation test scores and basal forebrain volumes revealed that only the mixed allo/egocentric computer-based navigation task was significantly correlated to posterior (Figure 4D; *R* = 0.31, *p* = 0.027; Table 3) and whole basal forebrain (*R* = 0.29, *p* = 0.037) volumes. This was in spite of the performances of subjects in the real space and computer-based versions of navigation task being significantly correlated (allocentric, *R* = 0.78, *p* < 0.001; egocentric, *R* = 0.79, *p* < 0.001; allo/egocentric, *R* = 0.84, *p* < 0.001; delayed, *R* = 0.41, *p* = 0.006).

**Table 3:** **Overview of the results for bivariate correlations between basal forebrain volumes and real/virtual navigation performance**.

	Real navigation								Virtual navigation							
	Whole cohort				AD				Whole cohort				AD			
	A	E	A/E	D	A	E	A/E	D	A	E	A/E	D	A	E	A/E	D
BF Anterior	*	–	**	–	–	–	**	–	–	–	–	–	–	–	–	–
BF Posterior	*	–	–	–	–	–	–	–	–	–	*	–	–	–	*	–
BF Whole	*	–	*	–	–	–	*	–	–	–	*	–	–	–	–	–

*BF, basal forebrain; AD, Alzheimer’s disease; A, allocentric; E, egocentric; A/E, mixed allo/egocentric; D, delayed*.

*–, Correlation was not significant*.

*  0.05, **  0.01.

*Results from whole cohort and AD group correlations are shown. NC and aMCI correlation results were omitted as no significant interactions were found. Basal forebrain volumes and navigation scores were corrected for age, education, and gender*.

### Basal forebrain volume correlates with navigation scores in AD patients

**Figure 5:**
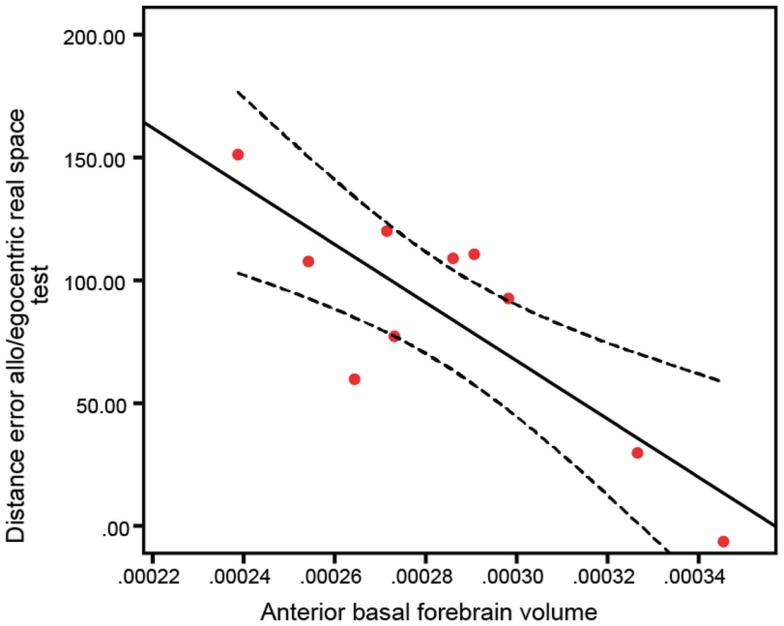
**Anterior basal forebrain volume of AD subjects is significantly correlated (*R* **=** 0.82, *p* **=** 0.003) to distance error in the mixed allo/egocentric real space test**. A linear fit line with 95% confidence interval is shown.

To test whether basal forebrain volumes were correlated to navigation dysfunction in clinical groups, we performed correlations of basal forebrain volumes and navigation task scores in individual groups. This analysis revealed no significant interactions of any basal forebrain volumetric measure with distance error in any of the real space or computer-based navigation tasks in the aMCI and NC groups. However, in the AD group, the anterior (Figure 5; *R* = 0.82, *p* = 0.003; Table 3) and whole (*R* = 0.68, *p* = 0.029) basal forebrain volumes significantly correlated with distance error in the mixed allo/egocentric real space task. In addition, posterior basal forebrain volume was significantly correlated to distance error in the mixed allo/egocentric virtual space task (*R* = 0.55, *p* = 0.040; Table 3).

### Regression analysis in the whole cohort and in the AD group

Basal forebrain measures (anterior and posterior) that showed significant correlations with real space navigation scores were entered into regression models with and without hippocampal volume to determine their relative contributions in predicting performance in the allocentric and mixed allo/egocentric navigation tasks for the whole cohort, and mixed allo/egocentric navigation performance for the AD patients.

When mixed allo/egocentric navigation performance for the whole cohort was considered as the dependent variable and each modality (anterior basal forebrain volume and hippocampal volume) was entered separately into the model, the relationship was significant for each modality (Table 4, Model 1). Both modalities were then entered together into the model to determine whether each modality independently contributed to mixed allo/egocentric navigation deficits when taking into account the effect of another modality (Table 4, Model 2). Although this model was still found to be predictive of mixed allo/egocentric navigation scores, neither hippocampal nor basal forebrain volume remained significantly associated with navigation performance.

**Table 4:** **Results of the regression models with distance error in the allo/egocentric real space test as the dependent variable and anterior basal forebrain volume and hippocampal volume separately (Model 1) or simultaneously (Model 2) entered as predictive variables, while controlling for age, gender, and years of education, within the whole cohort**.

Model	Volume	*R*^2^	Beta	*t*-value	*p*-value
1	BF anterior Hippocampus	0.14** 0.17**	−0.378 −0.414	−2.738 −3.047	0.009 0.004
2	BF anterior +Hippocampus	0.20**	−0.210 −0.293	−1.272 −1.780	0.21 0.082

*R*^2^ = *R*^2^ value of the overall model significant at **p < 0.01. Beta = standardized regression coefficient. *p*-value = association of each modality with the dependent variable. BF, basal forebrain.

When allocentric navigation performance in the whole cohort was considered as the dependent variable and each modality (anterior and posterior basal forebrain volume and hippocampal volume) was entered separately into the model, relationships were again significant for all modalities (Table 5, Model 1). However, when entering combinations of two modalities into the model (Table 5, Model 2), only those combinations including hippocampal volume, but not a combination of anterior and posterior basal forebrain volume, showed significant association with allocentric test scores. When entering both hippocampal volume and basal forebrain volume into the model, only hippocampal volume remained significantly associated with allocentric navigation performance. By contrast, when mixed allo/egocentric navigation performance in the AD group was considered as the dependent variable and each modality (anterior basal forebrain volume and hippocampal volume) was entered separately into the model, the relationship was only significant for anterior basal forebrain volume, but not hippocampal volume (Table 6, Model 1). Therefore, when entering both modalities together into the model, only anterior basal forebrain volume, but not hippocampal volume, remained significantly associated with mixed allo/egocentric navigation performance in the AD group.

**Table 5:** **Results of the regression models with distance error in the allocentric real space test as the dependent variable and anterior basal forebrain volume, posterior basal forebrain volume and hippocampal volume separately (Model 1) or simultaneously (Model 2 and Model 3) entered as predictive variables, while controlling for age, gender, and years of education, within the whole cohort**.

Model	Volume	*R*^2^	Beta	*t*-value	*p*-value
1	BF anterior BF posterior Hippocampus	0.10* 0.12* 0.26**	−0.316 −0.339 −0.512	−2.107 −2.279 −3.772	0.041 0.028 0.001
2	BF anterior + BF posterior	0.13	−0.232 −0.147	−1.056 −0.668	0.297 0.508
	BF anterior + Hippocampus	0.26**	0.011 −0.519	0.060 −2.931	0.952 0.006
	BF posterior + Hippocampus	0.27**	−0.064 −0.475	−0.383 −2.823	0.704 0.007
3	BF anterior + BF posterior + Hippocampus	0.27**	0.075 −0.106 −0.498	0.342 −0.507 −2.718	0.734 0.615 0.01

*R*^2^ = *R*^2^ value of the overall model significant at *p < 0.05, **p < 0.01. Beta = standardized regression coefficient. *p*-value = association of each modality with the dependent variable. BF, basal forebrain.

**Table 6:** **Results of the regression models with distance error in the allo/egocentric real space test as the dependent variable and anterior basal forebrain volume and hippocampal volume separately (Model 1) or simultaneously (Model 2) entered as predictive variables, while controlling for age, gender, and years of education, within the AD group**.

Model	Volume	*R*^2^	Beta	*t*-value	*p*-value
1	BF anterior Hippocampus	0.68** 0.183	−0.824 −0.428	−4.107 −1.339	0.003 0.217
2	BF anterior + Hippocampus	0.68*	−0.829 0.010	−3.284 0.041	0.013 0.969

*R*^2^ = *R*^2^ value of the overall model significant at *p < 0.05, **p < 0.01. Beta = standardized regression coefficient. *p*-value = association of each modality with the dependent variable. BF, basal forebrain.

## Discussion

Basal forebrain atrophy, particularly of the posterior nuclei, has previously been reported in aMCI and AD subjects (Grothe et al., 2011; Kerbler et al., 2014). Although in the current cohort of subjects basal forebrain volumes were not significantly reduced in the AD group compared to the NC group, atrophy of the basal forebrain, particularly the anterior area, was found to be significantly correlated to real space allocentric navigation, as well as to mixed allo/egocentric navigation in real space and computer-based tasks. No interactions with purely egocentric-based navigation performance were found. Furthermore, individual group correlations revealed a significant association of mixed allo/egocentric navigation performance with anterior basal forebrain volume in the AD group, an association that was independent of hippocampal change. This suggests that anterior basal forebrain degeneration is most closely associated with the navigation impairment observed in AD subjects. As we consistently found that significant interactions of navigation scores with whole basal forebrain volume could be explained by an interaction of navigation scores with a specific sub-region (anterior or posterior) of the basal forebrain, only results for anterior and posterior basal forebrain volumes are discussed below.

In particular, we demonstrated that the interaction between anterior basal forebrain volume and the performance of AD patients in the allocentric and mixed allo/egocentric navigation tasks was strong. By contrast, we did not find significant associations of basal forebrain volume with purely egocentric-type navigation. In the AD group, the significant correlations observed herein suggest that anterior basal forebrain degeneration reflects the observed functional decline in allocentric navigation performance in AD subjects. Regression analysis further revealed that the association of anterior basal forebrain volume with the AD-related decline in mixed allo/egocentric navigation ability was independent of hippocampal volume. Even though hippocampal volume was found to be significantly reduced in aMCI and AD subjects compared to the NC group, the regression model including the combination of both anterior basal forebrain volume and hippocampal volume as the predictor did not show an improvement of fit (or increase in R^2^) over using only the anterior basal forebrain as a modality. Our findings are consistent with previous studies in rodents that have demonstrated that basal forebrain-derived acetylcholine is important for tasks involving cues and cue detection including feature binding (Parikh et al., 2007; Botly and De Rosa, 2009), as well as a report that enhancement of cholinergic neuromodulation using physostigmine, an acetylcholine esterase inhibitor, increases the selectivity of neural responses during visual working memory tasks in humans (Furey et al., 2000).

Furthermore, the supply of acetylcholine to the hippocampus via the anterior Ch1–2 basal forebrain neurons is important for the plasticity of hippocampal place cells (Ikonen et al., 2002), and we have previously shown that the acetyl cholinesterase inhibitor donepezil improves navigation performance in the allocentric but not the egocentric hMWM task (Hort et al., 2014). Therefore, our work could provide additional evidence for the role of basal forebrain-supplied acetylcholine, particularly from the anterior nuclei, in allocentric spatial memory performance in humans.

Although basal forebrain volume and navigation performance were correlated in the AD group, and significant interactions were identified in the entire sample between basal forebrain volume and allocentric as well as mixed allo/egocentric test scores, no significant interactions were found between basal forebrain volume and navigation test scores in the other subgroups (NC and aMCI) when considered independently. A similar interaction has been reported for hippocampal volume (Nedelska et al., 2012). When considering the effect of hippocampal as well as basal forebrain volumes in the navigation performance of the entire cohort, the relationship of basal forebrain sub-region (anterior, posterior) or hippocampal volume with navigation in either task (allocentric, mixed allo/egocentric) became non-significant, suggesting that atrophy of these structures in cognitively normal and mildly impaired subjects might exert an effect only when they occur together, such that dysfunction of one cannot be compensated for by increased function of the other. In particular, cholinergic activity has been shown to be increased in the hippocampus of mild cognitively impaired subjects (DeKosky et al., 2002), suggesting compensatory increased activity of basal forebrain neurons in this condition. Indeed, a deterioration of the entorhinal–hippocampal pathway, which results in mild cognitive impairment (DeKosky et al., 2002; Ikonomovic et al., 2003), might cause increased sprouting of the basal forebrain–hippocampal connection (Stanfield and Cowan, 1982), which in turn could lead to a temporary increase in acetylcholine in the hippocampus (Contestabile, 2011). Coincident cholinergic and hippocampal degeneration would impair such compensatory mechanisms.

Anterior basal forebrain predicted performance independent of hippocampal volume in the AD group. One possibility is that allocentric and mixed allo/egocentric navigation impairment manifests only when both hippocampal and basal forebrain functions are compromised, with anterior basal forebrain degeneration occurring predominantly in the transition between mild and late-stage AD (Grothe et al., 2011). Therefore, in the regression analysis, anterior basal forebrain degeneration would be the most predictive element of early cognitive dysfunction, measured herein as allocentric navigation performance. Given that many aMCI subjects respond to cholinergic treatments and already have cholinergic dysfunction that may not fully be reflected by measures of basal forebrain volume loss, functional assessment of basal forebrain activity (e.g., cholinergic activity; Kim et al., 2012; Petrou et al., 2014) may provide increased sensitivity as a predictor of navigation performance of normal and aMCI subjects, compared to mere volumetric assessment.

Surprisingly, the basal forebrain volume was more strongly associated with real space navigation than navigation performance in the computerized task. Even though real space and computer-based navigation scores are highly correlated for each test (allo-, ego-, mixed allo/egocentric) for the whole cohort, only posterior basal forebrain volume correlated to computer-based mixed allo/egocentric navigation performance. In rats, the posterior basal forebrain – the nucleus basalis of Meynert, which projects cortically – is implicated in visual search (Botly and De Rosa, 2012), whereas cholinergic cells located in the anterior medial septum release acetylcholine in the hippocampus after stimulation of the vestibular (motion detection) system (Tai and Leung, 2012). Therefore, our finding that performance on the mixed allo/egocentric computer-based navigation task was significantly correlated to posterior basal forebrain atrophy would be consistent with the idea that a purely visual task such as the computer-based version of the hMWM is more dependent on the function of the nucleus basalis of Meynert, whereas the real space hMWM, which involves integration of vestibular as well as visual information, requires functional septal and nucleus basalis of Meynert basal forebrain neurons.

Although our study design cannot provide evidence of causality, the observed correlations are consistent with the basal forebrain being involved in the regulation of mixed allo/egocentric and allocentric, but not pure egocentric navigation in humans. In addition, atrophy of the basal forebrain may contribute to allocentric navigation impairments in subjects with severe dementia, independent of hippocampal changes. If longitudinal studies show this to be the case, our work would provide added justification for the potential use of the hMWM as a diagnostic tool for AD and/or aMCI subjects who may particularly benefit from drugs targeting the cholinergic system.

## Author Contributions

GK, JH, and EC designed the study. MV, JLi, and JLa conducted the experiments. JF, ZN, JLa, and GK analyzed the data. GK and EC wrote the manuscript with input from all authors. The study was managed by SR, JH, and EC.

## Funding Support

GK was funded by an ANZ Trustees PhD scholarship for medical research and a University of Queensland International Scholarship. EC was supported by a National Health and Medical Research Council of Australia Career Development Fellowship (569601). This project was funded by a ANZ Trustees Mason Foundation grant to EC, SR, and JH. ZN, JL, and JH received grant support from European Regional Development Fund–Project FNUSA-ICRC (CZ.1.05/1.1.00/02.0123) and project ICRC-ERA-HumanBridge (no. 316345); European Social Fund and the State Budget of the Czech Republic; European Social Fund within the project Young Talent Incubator II (reg. CZ.1.07/2.3.00/20.0117); Grant Agency of Charles University in Prague, grants 624012 and 546113; Ministry of Health, Czech Republic – conceptual development of research organization, University Hospital Motol, Prague, Czech Republic 00064203; Institutional Support of Laboratory Research Grant No. 2/2012 (699002); research projects AV0Z50110509 and RVO:67985823.

## Conflict of Interest Statement

Dr. Jan Laczó has consulted for Pfizer and was a shareholder of Polyhymnia-TS between June 2012 and May 2014. He declares that he has no other competing interests. Dr. Jakub Hort has consulted for Pfizer, Janssen, Merck, Novartis, Elan, Zentiva, and Ipsen and was a shareholder of Polyhymnia-TS between June 2012 and May 2014. Other coauthors declare that they have no commercial or financial relationships resulting in a conflict of interest.

## Abbreviations

AD, Alzheimer’s disease; AIBL, Australian Imaging, Biomarkers and Lifestyle study; aMCI, amnestic mild cognitive impairment; BF, basal forebrain; CSF, cerebrospinal fluid; GM, gray matter; hMWM, human Morris water maze; MMSE, mini-mental state examination; MPM, maximum probability map; MRI, magnetic resonance imaging; NC, normal controls; WM, white matter.

## References

[B1] Astur R. S. Taylor L. B. Mamelak A. N. Philpott L. Sutherland R. J. (2002). Humans with hippocampus damage display severe spatial memory impairments in a virtual Morris water task. Behav. Brain Res. 132, 77–84. 10.1016/S0166-4328(01)00399-0 11853860

[B2] Avants B. B. Epstein C. L. Grossman M. Gee J. C. (2008). Symmetric diffeomorphic image registration with cross-correlation: evaluating automated labeling of elderly and neurodegenerative brain. Med. Image Anal. 12, 26–41. 10.1016/j.media.2007.06.004 17659998 PMC2276735

[B3] Avants B. B. Yushkevich P. Pluta J. Minkoff D. Korczykowski M. Detre J. (2010). The optimal template effect in hippocampus studies of diseased populations. Neuroimage 49, 2457–2466. 10.1016/j.neuroimage.2009.09.062 19818860 PMC2818274

[B4] Berger-Sweeney J. Stearns N. A. Murg S. Floerke-Nashner L. R. Lappi D. A. Baxter M. G. (2001). Selective immunolesions of cholinergic neurons in mice: effects on neuroanatomy, neurochemistry, and behavior. J Neurosci 21, 8164–8173. 11588189 10.1523/JNEUROSCI.21-20-08164.2001 PMC6763842

[B5] Botly L. C. De Rosa E. (2009). Cholinergic deafferentation of the neocortex using 192 IgG-saporin impairs feature binding in rats. J. Neurosci. 29, 4120–4130. 10.1523/JNEUROSCI.0654-09.2009 19339607 PMC6665388

[B6] Botly L. C. De Rosa E. (2012). Impaired visual search in rats reveals cholinergic contributions to feature binding in visuospatial attention. Cereb. Cortex 22, 2441–2453. 10.1093/cercor/bhr331 22095213

[B7] Bourgeat P. Chetelat G. Villemagne V. L. Fripp J. Raniga P. Pike K. (2010). Beta-amyloid burden in the temporal neocortex is related to hippocampal atrophy in elderly subjects without dementia. Neurology 74, 121–127. 10.1212/WNL.0b013e3181c918b5 20065247

[B8] Butler T. Blackmon K. Zaborszky L. Wang X. Dubois J. Carlson C. (2012). Volume of the human septal forebrain region is a predictor of source memory accuracy. J. Int. Neuropsychol. Soc. 18, 157–161. 10.1017/S1355617711001421 22152217 PMC3339258

[B9] Contestabile A. (2011). The history of the cholinergic hypothesis. Behav. Brain Res. 221, 334–340. 10.1016/j.bbr.2009.12.044 20060018

[B10] DeKosky S. T. Ikonomovic M. D. Styren S. D. Beckett L. Wisniewski S. Bennett D. A. (2002). Upregulation of choline acetyltransferase activity in hippocampus and frontal cortex of elderly subjects with mild cognitive impairment. Ann. Neurol. 51, 145–155. 10.1002/ana.10069 11835370

[B11] Furey M. L. Pietrini P. Haxby J. V. (2000). Cholinergic enhancement and increased selectivity of perceptual processing during working memory. Science 290, 2315–2319. 10.1126/science.290.5500.2315 11125148

[B12] George S. Mufson E. J. Leurgans S. Shah R. C. Ferrari C. Detoledo-Morrell L. (2011). MRI-based volumetric measurement of the substantia innominata in amnestic MCI and mild AD. Neurobiol. Aging 32, 1756–1764. 10.1016/j.neurobiolaging.2009.11.006 20005600 PMC2888825

[B13] Grothe M. Heinsen H. Teipel S. (2012). Longitudinal measures of cholinergic forebrain atrophy in the transition from healthy aging to Alzheimer’s disease. Neurobiol. Aging 43, 1210–1220. 10.1016/j.neurobiolaging.2012.10.018 23158764 PMC4058576

[B14] Grothe M. Heinsen H. Teipel S. J. (2011). Atrophy of the cholinergic basal forebrain over the adult age range and in early stages of Alzheimer’s disease. Biol. Psychiatry 71, 805–813. 10.1016/j.biopsych.2011.06.019 21816388 PMC3701122

[B15] Hamlin A. S. Windels F. Boskovic Z. Sah P. Coulson E. J. (2013). Lesions of the basal forebrain cholinergic system in mice disrupt idiothetic navigation. PLoS ONE 8:e53472. 10.1371/journal.pone.0053472 23320088 PMC3540070

[B16] Hasselmo M. E. McGaughy J. (2004). High acetylcholine levels set circuit dynamics for attention and encoding and low acetylcholine levels set dynamics for consolidation. Prog. Brain Res. 145, 207–231. 10.1016/S0079-6123(03)45015-2 14650918

[B17] Hort J. Andel R. Mokrisova I. Gazova I. Amlerova J. Valis M. (2014). Effect of donepezil in Alzheimer disease can be measured by a computerized human analog of the Morris water maze. Neurodegener. Dis. 13, 192–196. 10.1159/000355517 24192578

[B18] Hort J. Laczo J. Vyhnalek M. Bojar M. Bures J. Vlcek K. (2007). Spatial navigation deficit in amnestic mild cognitive impairment. Proc. Natl. Acad. Sci. U.S.A. 104, 4042–4047. 10.1073/pnas.0611314104 17360474 PMC1820705

[B19] Ikonen S. Mcmahan R. Gallagher M. Eichenbaum H. Tanila H. (2002). Cholinergic system regulation of spatial representation by the hippocampus. Hippocampus 12, 386–397. 10.1002/hipo.1109 12099489

[B20] Ikonomovic M. D. Mufson E. J. Wuu J. Cochran E. J. Bennett D. A. Dekosky S. T. (2003). Cholinergic plasticity in hippocampus of individuals with mild cognitive impairment: correlation with Alzheimer’s neuropathology. J. Alzheimers Dis. 5, 39–48. 12590165 10.3233/jad-2003-5106

[B21] Kerbler G. M. Fripp J. Rowe C. Villemagne V. L. Salvado O. Rose S. (2014). Basal forebrain atrophy correlates with amyloid β burden in Alzheimer’s disease. Neuroimage Clin. 7, 105–113. 10.1016/j.nicl.2014.11.015 25610772 PMC4299972

[B22] Kim M. J. Lee K. M. Son Y. D. Jeon H. A. Kim Y. B. Cho Z. H. (2012). Increased basal forebrain metabolism in mild cognitive impairment: an evidence for brain reserve in incipient dementia. J. Alzheimers Dis. 32, 927–938. 10.3233/JAD-2012-120133 22903128

[B23] Laczo J. Andel R. Vlcek K. Macoska V. Vyhnalek M. Tolar M. (2011). Spatial navigation and APOE in amnestic mild cognitive impairment. Neurodegener. Dis. 8, 169–177. 10.1159/000321581 21124005

[B24] McKhann G. Drachman D. Folstein M. Katzman R. Price D. Stadlan E. M. (1984). Clinical diagnosis of Alzheimer’s disease: report of the NINCDS-ADRDA Work Group under the auspices of Department of Health and Human Services Task Force on Alzheimer’s Disease. Neurology 34, 939–944. 10.1212/WNL.34.7.939 6610841

[B25] Morris J. C. (1993). The clinical dementia rating (CDR): current version and scoring rules. Neurology 43, 2412–2414. 10.1212/WNL.43.11.2412-a 8232972

[B26] Mufson E. (2003). Human cholinergic basal forebrain: chemoanatomy and neurologic dysfunction. J. Chem. Neuroanat. 26, 233–242. 10.1016/s0891-0618(03)00068-1 14729126

[B27] Nedelska Z. Andel R. Laczo J. Vlcek K. Horinek D. Lisy J. (2012). Spatial navigation impairment is proportional to right hippocampal volume. Proc. Natl. Acad. Sci. U.S.A. 109, 2590–2594. 10.1073/pnas.1121588109 22308496 PMC3289383

[B28] Parikh V. Kozak R. Martinez V. Sarter M. (2007). Prefrontal acetylcholine release controls cue detection on multiple timescales. Neuron 56, 141–154. 10.1016/j.neuron.2007.08.025 17920021 PMC2084212

[B29] Petersen R. C. (2004). Mild cognitive impairment as a diagnostic entity. J. Intern. Med. 256, 183–194. 10.1111/j.1365-2796.2004.01388.x 15324362

[B30] Petrou M. Frey K. A. Kilbourn M. R. Scott P. J. Raffel D. M. Bohnen N. I. (2014). In vivo imaging of human cholinergic nerve terminals with (-)-5-(18)F-fluoroethoxybenzovesamicol: biodistribution, dosimetry, and tracer kinetic analyses. J. Nucl. Med. 55, 396–404. 10.2967/jnumed.113.124792 24481024

[B31] Sarter M. Gehring W. J. Kozak R. (2006). More attention must be paid: the neurobiology of attentional effort. Brain Res. Rev. 51, 145–160. 10.1016/j.brainresrev.2005.11.002 16530842

[B32] Schliebs R. Arendt T. (2006). The significance of the cholinergic system in the brain during aging and in Alzheimer’s disease. J. Neural Transm. 113, 1625–1644. 10.1007/s00702-006-0579-2 17039298

[B33] Stanfield B. B. Cowan W. M. (1982). The sprouting of septal afferents to the dentate gyrus after lesions of the entorhinal cortex in adult rats. Brain Res. 232, 162–170. 10.1016/0006-8993(82)90619-9 7055693

[B34] Tai S. K. Leung L. S. (2012). Vestibular stimulation enhances hippocampal long-term potentiation via activation of cholinergic septohippocampal cells. Behav. Brain Res. 232, 174–182. 10.1016/j.bbr.2012.04.013 22521836

[B35] Weniger G. Ruhleder M. Wolf S. Lange C. Irle E. (2009). Egocentric memory impaired and allocentric memory intact as assessed by virtual reality in subjects with unilateral parietal cortex lesions. Neuropsychologia 47, 59–69. 10.1016/j.neuropsychologia.2008.08.018 18789955

[B36] Zaborszky L. Hoemke L. Mohlberg H. Schleicher A. Amunts K. Zilles K. (2008). Stereotaxic probabilistic maps of the magnocellular cell groups in human basal forebrain.NeuroImage 42, 1127-1141 10.1016/j.neuroimage.2008.05.055 18585468 PMC2577158

